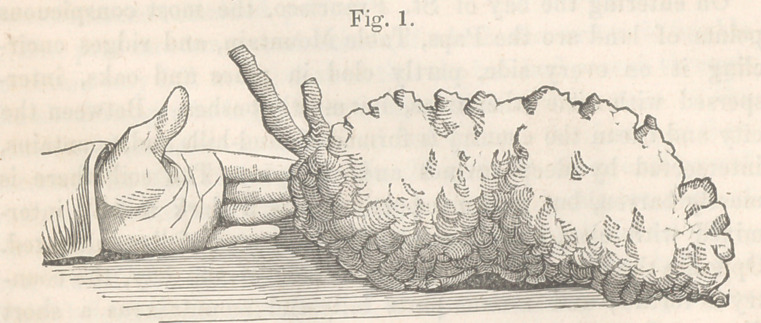# Remarks on California

**Published:** 1851-01

**Authors:** G. R. B. Horner

**Affiliations:** Late Fleet Surgeon, Pacific Squadron


*Remarks on California.* By G. R. B. Horner, M. D., U. S. N., late Fleet Surgeon, Pacific Squadron.

Though many letters have been written concerning the above country, but few have contained medical information—and this has come mostly from unprofessional sources. The letters written have been generally about the gold mines, and have imparted little knowledge respecting the climate, productions of the soil, or other subjects calculated to interest a physician or other persons wishing for information, special or general. I shall then endeavor to present such as I was able to collect during my sojourn there, which was from August, 1849, to the middle of last July. More than half that period was passed at Benicia, and a greater part of the remainder at, and in the vicinity, of St. Francisco. A short time was spent in travelling to the northern mining region. My public duties in our squadron, the great difficulty in procuring means of conveyance from many points to others, and the enormous expense attending it, prevented me from making as minute or general observations upon the country as I desired. Such, however, as seem worthy of it, I will now make known.

On entering the bay of St. Francisco, the most conspicuous points of land are the Paps, Table Mountain, and ridges encircling it on every side, partly clad in pines and oaks, interspersed with some other trees, but mostly bushes. Between the city and ocean the country is formed of sand-hills and mountains, intersected by deep ravines and valleys. The soil there is mostly barren, but in some portions has a dark mould, intermixed with clay, and is productive, when carefully cultivated. Opposite the city, and on the eastern side of the bay, the country is fertile; and in some parts hilly and mountainous a short distance from the water. At the back of the ridge near it, may be seen the top of the Devil's mountain, the highest one in that portion of the country. It rises nearly four thousand feet above the ocean, and is one of the best land marks for vessels plying up and down the bay, and the two great rivers, discharging into the most eastern part of it, termed the bay of Suison. These rivers are the Sacramento and St. Joaquim. The first runs nearly south, the second to the north.

Both derive their chief sources from the western face of the Sierra Nevada, overflow their banks, and are bordered by an exceedingly rich, alluvial soil. The banks of the Sacramento, I found in July, when the water was of medium depth, not more than twenty feet above it on either side, and covered almost without interruption, for ninety miles above its mouth, with a dense growth of cotton wood, oaks, willows, sycamores, and bushes. Back of the woods are plains of great extent, which are inundated in winter. Marshes, called tulas, exist in certain parts, and form islands of great extent, where the two rivers disembogue. North and south of that portion of the bay designated the straits of Karquines, which lie between the bay of Suison and St. Paul's, or the north-western part of that of St. Francisco, the country is formed of lofty, rounded hills. Those on the northern side are clad with a luxuriant crop of wild oats during the spring and summer, and those on the southern side are covered with them, and a scattered growth of low wide-spreading horse-chesnuts and oaks, mingled with some elders and sycamores on the low grounds and along the banks of streams. Fennel, a bulbous plant, used for washing, a very large tuberous root, (fig. 1) belonging to a vine called wild cucumber, like

Fig. 1.

a sweet potatoe, the endive, wild artichoke, and a multitude of flowers, are likewise productions. Of the oaks there are several kinds, the most remarkable of which are those bearing an acorn two or more inches long, and a dwarf oak noted for its exceedingly poisonous effects. By contact, and it is believed, in certain cases, by merely allowing the effluvia from it to touch the skin, these effects are produced. I saw an officer of our squadron with his face so swelled that he could hardly see, and yet I understood from him he had not touched the plant, and could have been poisoned only by lying upon the ground where it was growing. This oak is a very small bush, bearing a reddish, somewhat serrate, and oval leaf, quite as large as those of the other oaks. Their foliage is very deficient, the nerves of every leaf end in points, forming quite sharp prickles, which are undoubtedly owing to the great drought of every year, parching the leaves, and taking away all moisture from the ground. So completely dried is the soil, that on hills, in plains and valleys, it cracks in every direction for one or more inches in width, and several feet in depth. The richer the soil, the more thoroughly it has been soaked by the rains of fall and winter, the more it bakes and fissures. When this begins to occur, grass, wild oats, and flowers begin to wither, and by July have changed their rich color to a pale yellow. Springs then become dry, brooks and rivulets disappear, and fires begin to sweep over the prairies and tulas, borne on the strong westerly breezes, which prevail from spring until late in the fall. When these cease, the easterly and northerly begin to blow, and continue at intervals until spring. When the wind is north-easterly the weather is serene and delightful, and it blows moderately, but when it becomes south-easterly and south-westerly it is squally, often violent, and accompanied with a deluge of rain. As this falls, vegetation revives, grass and oats spring forth, and flowers begin to enamel the fields. Even in the dead of winter they flourish on hills and plains, and during the last it was a singular sight to see the former clothed with the richest verdure and capped twice by resplendent snow. As vegetation thrives, the innumerable bands of cattle fatten, elk, deer, and antelopes quit the low grounds and range freely over the hills, the prairie wolf or kiote gluts himself more luxuriously on sheep and other small animals, and the grisly bear, the greatest terror of the country, has a choice of flesh or vegetables. Esculent fruits of every kind are rare. Scarcely one is to be found at any season, and a few peaches, apples, grapes, oranges from foreign regions, and wild berries, were all I saw. Vegetables for culinary purposes were principally imported: potatoes, Irish and sweet, pumpkins and onions were sold at enormous prices. At twenty cents a pound the former were cheap, and at Marysville, near the upper mines, the latter were sold for $1.25. These prices were owing mainly to the great majority of persons being employed in the gold mines. A few attempted cultivation, and were quite successful when irrigation was used, but from a want of a proper knowledge of the seasons, and neglecting to seed soon after the rains began, most persons were only partially successful. The farmers had likewise to contend with the swarms of grasshoppers infesting the country, and I heard of one garden, on the straits mentioned, being desolated by these insects within a few hours. They will continue to be destructive to gardens and farms until some method of destroying them is discovered. But they are not useless; they are killed, dried, and eaten by the Indians. Of this fact I was informed by several credible persons, and by one of the overland emigrants, who had found vast quantities in some of their huts. Acorns, and the kernel of a very large pine cone, are said also to be used for food by them.

For more definite remarks on the climate I refer to the following abstract of a register kept strictly by myself. I regret that the register is too large and minute to insert in this journal, but the abstract will serve to give a general knowledge of the climate.

| 1849 | Thermometer | Barometer. | Hygrometer. | Wind. | Rainy days. | General Remarks. |
|---|---|---|---|---|---|---|
| August. | | | | | | Ship off San Francisco. The register includes only eight days in California, or those after she made the coast on the 24th of August. All of them were misty. |
| Average. | 59 | 30.04 | 30⅓ | West- | 0 | |
| Maximum. | 63 | 30.10 | 34 | erly. | | |
| Minimum. | 55 | 29.89 | 26 | | | |
| September. | | | | | | In September 20 misty and cloudy days, and 10 fair. Anemometer 3 seconds. |
| Average. | 57⅔ | 30.09¾ | 26 | 20 Sy. | 0 | |
| Maximum. | 67 | 30.20 | 34 | 4 Ny. | | |
| Minimum. | 61 | 29.94 | 20 | 6 var. | | |
| October. | | | | | | Fair eight days. Heavy constant rains on the 10th 22 days misty, cloudy and variable. Anemometer as high as 3 seconds and as low as 14. |
| Average. | 62⅔ | 30.06 | 25⅓ | 11 Ny. | 1 | |
| Maximum. | 73 | 30.25 | 32 | 17 Sy. | | |
| Minimum. | 52½ | 29.85 | 16 | 4 var. | | |

| 1849 | Thermometer | Barometer. | Hygrometer. | Wind. | Rainy days. | General Remarks. |
|---|---|---|---|---|---|---|
| November. | | | | | | |
| Average. | 54 | 30.08 | 24 | 12 Ny. | 10 | Ship at Saucelito, 5 miles from city. Six fair days, 14 misty. Anemometer variable from 3 to 10 seconds. Calm sometimes. |
| Maximum. | 65 | 30.35 | 30 | 17 Sy. | | |
| Minimum. | 52 | 29.55 | 16 | 16 var. | | |
| December. | | | | | | Snow fell on the 3d and covered the adjacent mountains. Seven fair days, 25 rainy, misty, and variable. Hail fell on the 14th. Storm on 3 distinct days, thunder and lightning on one. Anemometer 4 seconds during them. |
| Average. | 50½ | 30.04½ | 22¾ | 10 Ny. | 12 | |
| Maximum. | 57 | 30.30 | 30 | 14 Sy. | | |
| Minimum. | 40 | 29.55 | 16 | 7 var. | | |
| 1850 Jan | | | | | | Only 5 fair days, 26 misty, cloudy, and rainy. Ship went up to Benicia, 30 miles above St. Francisco, and remained there until after I left her for home. Anem. 3 seconds on the 9th. |
| Average. | 50 | 30.04⅔ | 22⅔ | 7 Ny. | 15 | |
| Maximum. | 62 | 30.25 | 27 | 15 Sy. | | |
| Minimum. | 40 | 29.85 | 19 | 9 var. | | |
| February. | | | | | | No rain fell until the 21st, we then had it for 4 days at intervals. On the 27th snow fell and covered the hills, but melted in a day or two. The Devil's and other mountains remained covered with it for weeks. During a squall the anemometer was 3 seconds, generally was much less. |
| Average. | 48½ | 30.13¼ | 22¼ | 8 Ny. | 4 | |
| Maximum. | 57 | 30.36 | 26 | 26 Sy. | | |
| Minimum. | 40 | 29.55 | 19 | 5 var. | | |
| March. | | | | | | |
| Average. | 56⅔ | 30.10⅓ | 24⅔ | 10 Ny. | 8 | Rain fell in light showers. Wind squally, but not violent at other times. Anemometer 4 to 14 seconds. |
| Maximum. | 72 | 30.35 | 33 | 11 Sy. | | |
| Minimum. | 40 | 29.75 | 19 | 10 var. | | |
| April. | | | | | | |
| Average. | 56 | 30.09½ | 26⅔ | 10 W. | 3 | Rain fell last on the 19th, and in light showers. Eighteen days were fair and pleasant. Highest range of anemometer 4. |
| Maximum. | 71 | 30.25 | 34 | 9 N W | | |
| Minimum. | 47 | 29.95 | 18 | 8 S. | | |

| 1850 | Thermometer | Barometer. | Hygrometer. | Wind. | Rainy days. | General Remarks. |
|---|---|---|---|---|---|---|
| May. | | | | | | Twenty-five days fair. A halo about the sun on the 10th, slight mist for several days. Anemometer ranged from 4 to 20 seconds. |
| Average. | 61¼ | 30.01 | 26⅔ | 9 S W | 0 | |
| Maximum. | 71 | 30.14 | 34 | 10 NW | | |
| Minimum. | 52 | 29.85 | 23 | 12 W. | | |
| June. | | | | | | Twenty seven days were fair, but a few were slightly misty. Wind moderate. Anemometer not above 4 and as low as 20 seconds. |
| Average. | 62 | 30.00⅓ | 26⅓ | 12 S W | 0 | |
| Maximum. | 76 | 30.19 | 29 | 9 W. | | |
| Minimum. | 54 | 29.85 | 22 | 9 N W. | | |

On the first of July I left the ship for the mines, and could not keep the register with regularity, but I will state that the weather was excessively hot at them, and so cold and damp at St. Francisco, that fire was indispensable for comfort. On the 13th, at 8 o'clock, P. M., Fah.'s thermometer stood at 52° in the latter place, and at Long Bar, upon the 4th of July, it stood in the afternoon at 106°. In Marysville, twenty miles below, and at the junction of the Yuba with Feather river, the heat was 112°. This was accompanied by a corresponding dryness of the air and earth, except on the low grounds near the former river.

In making observations on the force of the wind, the Anemometer or ventometer used was one of my own contrivance, caused by the want of any other instrument of the kind during our long passage around Cape Horn. My instrument consists of a wind-mill wheel fixed in the middle of an axle, beneath the back part of which is placed a reel for a silk line thirty feet long. The axle of each turns on pivots, and during the revolutions of the wheel the line is transferred from the reel to the axle. When this has been done the line is restored to its place by means of a wince connected with the axle of the reel, and taken off at the time of experimenting. The above anemometer I found very sensitive to the lightest winds, and it enabled me very accurately to determine their relative force, but for this purpose I used sometimes another instrument formed of a wheel and axle, similar to that of the first one, and having two cog wheels connected with a face like that of a clock, and fixed in front of a house-like box. The smaller cog wheel has four teeth, the largest sixty-four. This has the hand of the face attached to the outer end of its axle. The hand indicates the number of revolutions of the wind-wheel in any given time, and the precise number may be ascertained by multiplying their number by four, the number of the teeth to the smaller cog wheel affixed to the posterior end of the axle to the above wheel.

As winter approaches, rain falls and vegetation revives, game becomes more abundant, wild ducks and geese flock in vast numbers upon the ponds, inlets, bays, rivers and creeks. Among other birds found at that season, and during the whole year, are snipe, curlews, cranes, bitterns, buzzards, crows, avosets, grebes, pelicans, cormorants, top-knotted partridges, magpies, and sparrows. The chief birds of prey are hawks, the condor, owls, and white headed eagles, which may be seen flying over the Sacramento in quest of prey, or quietly perched on the tops of the adjacent trees. At all seasons an abundance of fish may be procured by hauling a seine in various parts of the bay of St. Francisco. The fish commonly caught are sprats, perch, mullets, eels, trout, crabs, and sturgeons of fine esculent qualities. Well cooked, their flesh is white, tender, and very savory. In some parts excellent salmon are caught plentifully, and when the Californians can turn their attention from the search after gold, to that of their bays and rivers, no doubt can exist that they will yield as many fish as they can consume.

Of the mineral productions of the country my limited opportunities of research will not allow of my speaking at large; and I will merely remark, that besides all attractive gold, it yields iron ore, cinnabar, jasper, carbonate of magnesia, lime and various petrifactions. Near Benicia a yellowish, soft sand stone exists in immense quantities. It is well adapted for buildings, and notwithstanding the high wages of workmen, is now used by them for their foundations. Some miles northward of the town are several sulphur springs. They arise at the foot of Red mountain, are copious, and already resorted to by invalids. In the waters of one of the springs, I found, on analysis, some magnesia, and a large amount of the muriate of soda—besides the sulphur—and sulphuretted hydrogen, which was constantly bubbling up from the bottom.

(To be concluded in our next.)

## Figures and Tables

**Fig. 1. f1:**